# Conformity and individual preference shape nest material use in zebra finches (*Taeniopygia guttata*)

**DOI:** 10.1371/journal.pone.0342277

**Published:** 2026-02-11

**Authors:** Julia L. Self, Andrés Camacho-Alpízar, Priya Binwal, Umama Mir, Stefan Stanescu, Benjamin A. Whittaker, Lauren M. Guillette

**Affiliations:** Department of Psychology, Animal Cognition Research Group, University of Alberta, Edmonton, Alberta, Canada; McGill University, CANADA

## Abstract

Culture, learned behaviours shared within a group, is found in diverse species and critically impacts fitness. Culture arises through social learning and persists when newcomers adopt group norms via conformity and conformist transmission. We examined conformity and conformist transmission in a novel context: avian nest building, testing whether male zebra finches, the primary nest builders in this species, forgo their preferred nest material colour in favour of the majority-demonstrated alternative, and whether the likelihood of switching was disproportionate relative to majority size. First, we determined each male’s preferred colour and preference strength. Then, each male, paired with a female, was introduced into a population containing four conspecific pairs, each incubating eggs in a nest. Populations varied in the number of nests (0–4) built using the male’s non-preferred colour material. After three days, males were given both material colours for nest construction. Males with weaker preferences were more likely to conform, using the majority-demonstrated, non-preferred colour, whereas males with stronger preferences resisted social influence. Although males often acquired social information – evidenced by their initial interactions with the majority-demonstrated material – this did not consistently translate into conformist nest-building behaviour. These findings show socially influenced nest-building and individual variation in susceptibility to social information.

## Introduction

Culture – defined as socially learned behaviours that are shared among members of a population and persist over time [1] – is central to human life, shaping everything from diet and language to art and spirituality [2]. Although once considered to be uniquely human, cultural behaviours in nonhuman animals (“animals”, hereafter) were first scientifically documented in the mid-20^th^ century through observations such as milk-bottle opening in birds and sweet potato washing in monkeys [3,4]. However, pre-Western Indigenous knowledge systems and oral traditions had long recognized such behaviours as cultural [5]. Over the past seven decades, research examining animal culture has flourished, with evidence of cultural phenomena now reported across a wide range of taxa and behavioural domains. These include migratory traditions in bighorn sheep (*Ovis canadensis*) [6] and whooping cranes (*Grus americana*) [7]; vocal dialects in sparrows (*Zonotrichia leucophrys*; *Passerculus sandwichensis*) [8,9] and humpback whales (*Megaptera novaeangliae*) [10], and mating preferences in fruit flies (*Drosophila melanogaster*) [11] and reef fish (*Thalassoma bifasciatum*) [12]. Collectively, this body of research highlights culture as a second system of inheritance, alongside genetics, through which ecologically important behaviours and information are transmitted [13,14]. As culture is increasingly recognised as a factor shaping population dynamics, it has become a growing focus in conservation science [15]. Understanding the mechanisms that stabilise or erode cultural traditions and identifying the species and behavioural domains in which culture plays a role remains a critical and timely scientific challenge.

Culture arises through the transmission of behaviours via social learning [16] and is maintained over time through two key processes. The first, *conformity*, occurs when individuals with existing behavioural preferences forgo their own preferences by adopting a different behaviour that is preferred by the majority within a group [17–19]. The second, *conformist transmission*, occurs when naïve or inexperienced individuals disproportionately adopt the majority behaviour preferred by their group – that is, the proportion of new individuals who adopt the majority behaviour is larger than the proportional size of the majority [18,20,21]. The roles of both processes in maintaining cultural stability were demonstrated in a field study with great tits (*Parus major*) [16]. In this study, pairs of birds from neighbouring wild populations were trained to solve a novel foraging task by sliding open either the red or blue side of a feeder door access food. Upon release into their populations of origin, these seeded behaviours spread rapidly through each population via social learning, giving rise to stable local traditions. Naïve individuals showed *conformist transmission* by disproportionately adopting the most common behavioural variant in their population. Additionally, immigrants that originated from a population using one solution (e.g., red side of the door), switched their preference to match the dominant behaviour in their new population (e.g., blue side of the door), demonstrating *conformity* [16]. While conformity and conformist transmission are behavioural mechanisms that maintain culture, there are multiple different cognitive mechanisms and social learning strategies that can result in conformist patterns of copying (i.e., copy-the-majority, social facilitation [22,23]. See Table 1 for more information about conformity and conformist transmission.

**Table 1:** Summary of Conformity and Conformist transmission.

	Conformity	Conformist transmission
**Definition**	Discounting personal preference in favour of adopting the majority behaviour in a population (e.g., [17,18]).	Disproportionate adoption of the majority behaviour in a population (e.g., [20,21]).
**Assumptions**	Individual is: (1) Exposed to multiple behavioural variants, and, (2) Has a measured pre-existing preference for one variant over the other(s) [16,17,24].	Individual is: (1) Exposed to multiple behavioural variants, and (2) May or may not have a measured pre-existing preference for one variant over the other(s) [16,25,26].
**Methodology**	Measure a focal individual’s initial preference for the behavioural variants, then reassess their preference after exposure or relocation into a population whose majority demonstrates a different behavioural variant [16,17,24].	Measure a focal individual's preferred behavioural variant after exposure to a group demonstrating multiple behavioural variants, with one variant exhibited by the majority [16].
**Level of analysis**	Individual (e.g., an individual increases their use/reliance on the behavioural variant demonstrated by the majority) [17]	Population/Group (e.g., when the proportion of individuals adopting the majority behavioural variant is greater than the proportional size of the majority) [20].
**Consequences**	Increases within-group behavioural homogeneity, helps promote culture, but is not on its own sufficient to maintain culture [21].	Promotes within-group behavioural homogeneity; increases relative majority of behavioural variant; population becomes increasingly homogenous over time [20,27].

Although conformity and conformist transmission are known for their roles in stabilising cultural traditions, empirical evidence for both conformity and conformist transmission in animal populations is mixed and controversial [18]. For example, migrating wild vervet monkeys (*Chlorocebus pygerythrus*) conform to the dietary preferences of their new groups [24], whereas migrating captive chimpanzees (*Pan troglodytes*) do not conform [25]. Similarly in fish, nine-spine sticklebacks (*Pungitius pungitius*) adjust their feeding site preferences to align with their social group [26], whereas guppies (*Poecilia reticulata*) retain their original foraging site preferences [28]. Such variability may reflect differences in the type of social influence being studied (i.e., conformist transmission vs. conformity), differences in experimental design and methodologies [18], as well as differences in how ‘majority’ is operationalized [29]. Importantly, conformity is not a fixed strategy but is influenced by multiple factors, including the majority size [30] and relative benefit of the majority behaviour [31], whether an individual possesses relevant personal information [28,32], and the costs of asocial learning [33]. Despite growing interest in these cultural processes, most studies have focused on tool-use and foraging behaviours. Additional and broader investigations across different behavioural domains, species, and ecological contexts are needed to fully understand the conditions under which culture evolves and persists.

A promising behavioural domain for examining conformity and conformist transmission is construction behaviour, particularly nest building in birds [34]. There are several reasons to expect conformity in avian nest building. First, social learning, the prerequisite for conformity and culture, is met: birds socially learn about the materials with which to construct their nests [35–37], and behaviours that favour social learning are also expected to favour conformity [33]. Second, fieldwork suggests that certain bird species exhibit architectural traditions, evident both in the structure [38] and material composition [39–41] of nests. Third, nest building is an ecologically important task that is critical for reproductive success; nest morphology (e.g., size, shape), material composition, and location each influence fitness, making reliance on asocial learning (e.g., trial-and-error) particularly costly. Finally, traditions and culture have been documented in many avian species and behavioural domains, including in vocalizations [42], foraging [3,16], tool-use [43–45], migratory patterns [7], and interspecies communication [46]. Although interest in nest building as a model system in cognition, ecology and evolution is growing [47,48], to our knowledge, there are yet to be any laboratory studies examining conformity in nest building.

In the current study, we examine conformity in nest material use in zebra finches (*Taeniopygia guttata*), a small songbird native to Australia. Zebra finches are non-territorial and social: forming large groups, nesting and breeding opportunistically in close-proximity colonies, and gathering at social-hotspots [49]. This species constructs dome-shaped nests, with males primarily responsible for collecting and depositing nest materials [50]. Our aim was to examine both conformity and conformist transmission by testing whether zebra finches who are naïve to nest building would forgo their own individual colour preferences for nest material in favour of adopting the nest material colour used by the majority in their population (*conformity*). Furthermore, we assessed whether this tendency to adopt the majority preference is disproportionate relative to the majority size (*conformist transmission*). To test this, we established artificial populations of breeding birds that varied in the number of nests constructed from blue versus yellow material ( "population composition"  hereafter). Focal male zebra finches, with known nest-colour preferences for either blue or yellow material, and paired with a female partner, were introduced into these experimental populations. After exposure to the population, focal males underwent a final colour preference test, where they were provided with both colours of nest material and a nest cup to construct a nest. We recorded the colour of string focal males first interacted with and used to construct their nests.

## Materials and methods

### Subjects

One-hundred and eighty-six adult zebra finches (93 female; 93 male) were used in this experiment. Seventy-seven unique male-female pairs (154 individuals) participated in the experiment as observers. Sixteen unique male-female pairs (32 individuals) acted as initial demonstrators and did not serve as observers. Subsequent demonstrator pairs came from the pool of observers who had already completed the experiment. Demonstrator pairs were used between four and twelve times in total. To control for potential demonstrator effects, experimental populations were yoked such that populations with opposing majority preferences (0P-4N with 4P-0N, and 1P-3N with 3P-1N) were run concurrently and observed the same demonstrator pairs. Each observer was naïve with respect to nest building, and each demonstrator had previously constructed at least one nest.

Each of the birds used in this experiment were bred at the University of Alberta and housed in same-sex cages (165 × 66 × 184 cm) in a colony room prior to entering the experiment. Colony rooms were maintained under a 14:10 light-dark cycle, with temperature at ~22°C, and humidity at ~50%. Birds had *ad-libitum* access to mixed seed (Hagen Canada), demineralized water, crushed oyster shell and cuttlefish shell (CanadianLabDiet). Vitamin water (Hagen Canada) and fresh spinach were provided to the birds three times per week, and spray millet (Hagen Canada) was provided once per week. After completing this experiment, subjects were housed in a large free-flight aviary. Efforts to alleviate suffering included housing birds in an enriched environment (perches, roosting boxes, large space to fly) and reducing the number of birds used by reusing birds across multiple experiments. Once birds had reached their experimental endpoints, they were either adopted out as pets to community members, euthanized via either transcardial perfusion (in the case of birds used in terminal experiments) or decapitation. All husbandry and procedures complied with the Canadian Council of Animal Care Guidelines, and all protocols were approved by the University of Alberta Animal Care and Use Committee (AUP00002923).

### Populations

To test whether zebra finches conform to the nest material choices of an established population, each observer pair was randomly assigned to one of five experimental populations. Each population consisted of four male-female demonstrator pairs, with each pair incubating eggs in a nest. Populations varied in the number of demonstrator nests (out of four) built with the observer male’s preferred (P) versus non-preferred (N) nest material colour. The population compositions for each of the five experimental populations were as follows: 4P-0N, where all four nests were built using the observer male’s preferred colour; 3P-1N, where three nests were built using the observer male’s preferred colour and one with his non-preferred colour; 2P-2N, where two nests were built using the observer male’s preferred colour and two with his non-preferred colour; 1P-3N, where one nest was built using the observer male’s preferred colour and three with his non-preferred colour; and 0P-4N, where all four nests were built using the observer male’s non-preferred colour.

### Holding room

A holding room contained cages (100 cm × 50 cm × 50 cm; King Cages International LLC) in which observers were paired for a minimum of two days before starting the experiment to facilitate the formation of pair bonds. Each cage held one observer pair and was visually separated from all other cages. Each cage was also equipped with three in-cage cameras (mini-BNC cameras, OSY CAMS). Husbandry procedures and environmental conditions in the holding room were the same as in the colony room.

### Social information rooms

**Fig 1:**
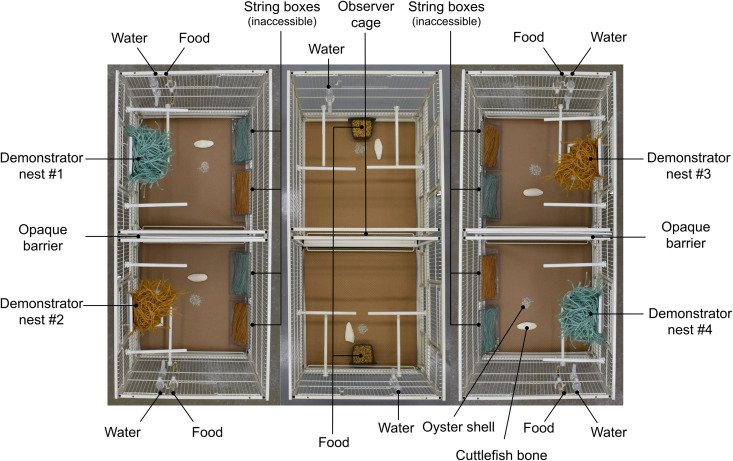
Top-down view of the social information room cages for the 2P-2N population. Each demonstrator cage-half (50 cm × 50 cm × 50 cm) contains one demonstrator pair incubating eggs in a nest, inaccessible blue and yellow string, food, water, and supplements (cuttlefish bone, oyster shell). The observer cage (100 cm × 50 cm × 50 cm) is in the centre of the two demonstrator cages and contains the observer pair, food, water, and supplements.

Each social information room housed one population and contained three cages (100 cm × 50 cm × 50 cm; King Cages International LLC) arranged side-by-side in a row. The centre of the three cages housed the observer pair and had a demonstrator cage on either side. Each demonstrator cage was divided in half by an opaque white plastic barrier, with one demonstrator pair housed in each half (four demonstrator pairs in total; Fig 1).

Each demonstrator pair received a white nest cup, positioned at the centre of the back wall of their half-cage so that the front of the nest cup was facing the observer cage, and 400 pieces of string with which they could construct a nest. In the laboratory, zebra finches readily construct nests using a variety of material types, including coloured string [35], coconut fibre [51], wood chips [52], wire [53], and strips of coloured paper [54]. To provide visual access to both colours of string, each demonstrator cage-half also contained two transparent boxes placed against the wall adjacent to the observer cage; one box contained 400 pieces of blue string and the other contained 400 pieces of yellow string. The string inside these boxes was inaccessible to the demonstrators but provided the observer with visual access to equal amounts of unused string of each colour within each demonstrator cage. We balanced the amount of unused string in the demonstrator cages as to ensure equivalent perceived availability of each colour and to avoid presenting observers with evidence of depletion (e.g., where there is less available string of the colour used to construct the nest). Previous work has shown that zebra finches may avoid copying the socially demonstrated behavioural variant, likely to avoid competition [55]. By having a large amount of both colours of string visible to the observers within each demonstrator cage during the social information phase, changes in colour preference can be attributed to social influence rather than lack of exposure to, or familiarity with, one of the colours compared to the other. Additionally, presenting observers with both the demonstrated and alternative option within each demonstrator cage gives the illusion that demonstrator birds chose the material with which they built, rather than using a material because it was the only one available. Exposing observers to both options (socially demonstrated, alternative) is thus standard in studies of conformity (e.g., [11,16,19,24–26]).

Experimental trials began once each of the four demonstrator pairs had constructed a nest and laid at least one egg. Demonstrator nests were checked daily for eggs, excluding days when observer birds were present in the room; real eggs were removed from the nest to prevent hatching of chicks, and were replaced with plastic zebra finch eggs (DummyEggs, Florida, USA). Each demonstrator nest had between one and five eggs in their nest when the observation phase began. While the number of eggs in the nest are unlikely to have been visible to the observers (eggs generally sit below the line of sight into the entrance hole), we required each demonstrator to have an egg in their nest to standardize the observation phase: each observer pair viewed demonstrator birds incubating eggs in nests. Husbandry procedures and environmental conditions were the same in the social information rooms as in the colony and holding rooms.

### Procedure

Each experimental trial consisted of three phases: (1) initial colour preference test, (2) observation phase, and (3) final colour preference test.

#### Phase 1: Initial colour preference test.

**Fig 2:**
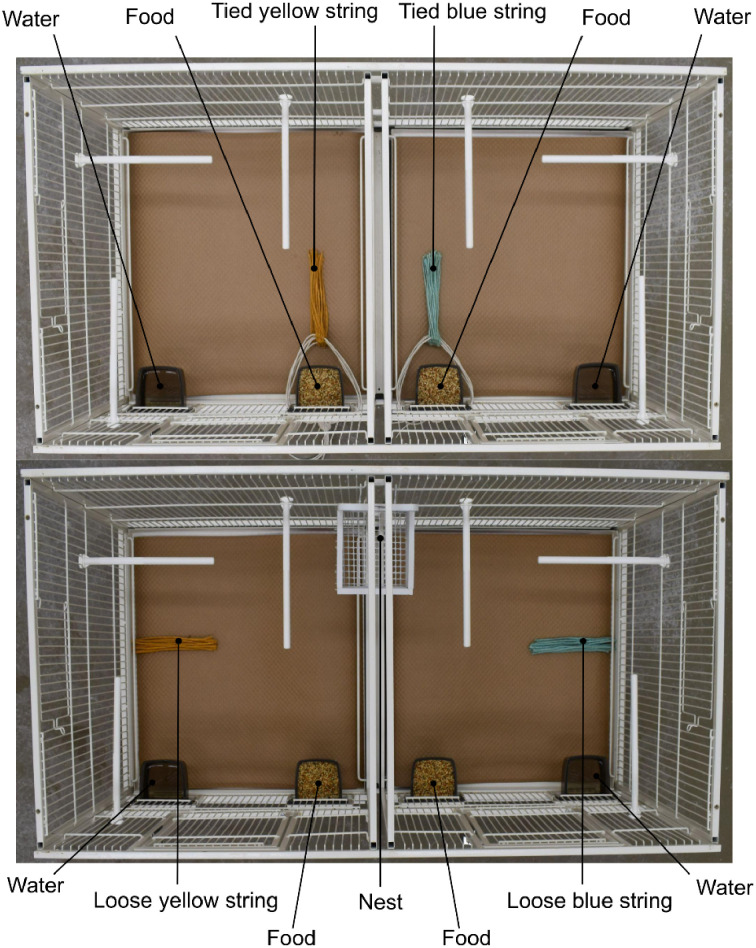
Top-down view of an observer’s holding room cage during the initial (top) and final (bottom) colour preference tests. During the initial colour preference test (top), 25-piece bundles of tied 15 cm blue and yellow string are placed inside the cage (100 cm × 50 cm × 50 cm) and tied to the cage wall. During the final preference test (bottom), 25-piece bundles of loose 15 cm blue and yellow string are placed inside the cage, on opposite sides of the cage floor, with each colour on the same side of the cage as in the initial colour preference test. A white nest cup is hung in the centre back of the cage for nest construction.

An observer’s initial colour preference test began two and a half hours after light onset in the holding room. To assess the birds’ colour preferences for potential nest materials, two bundles of string, one blue and one yellow (jute craft twine, James Lever Co., Bolton, UK), were placed on the floor of the observer cage. One string bundle was placed on the left side of the cage, and the other was placed on the right side of the cage (Fig 2; top panel); the side of the cage bundles were assigned to was randomized. Each bundle contained 25 pieces of 15 cm coloured strings; each coloured string was tied to a long white string which secured the string bundle to the inside wall of the observer cage and prevented the birds from using the string to construct a nest (Fig 2, top panel). String bundles remained tied inside the observer cage for four hours, at which point the bundles were removed.

Video of the observer pair interacting with each colour of string was recorded by in-cage cameras, and the total duration that the male observer (the primary nest builder of this species), spent interacting with each string colour was scored using BORIS Version 8.24.1 [56]. An ‘interaction’ was defined as any instance where the male observer touched the string bundle with any body part excluding the tail. We defined the observer male’s *initial colour preference* as the colour of string with which he interacted for the longest total duration and calculated his *preference strength* as the percentage of total string interaction time spent with his preferred colour. For instance, a male who spent 600 seconds interacting with yellow string and 400 seconds interacting with blue string would have an initial colour preference for yellow and would have a preference strength of 60%. This measure of initial preference strength is repeatable within-individuals for up to two months (well beyond the length of this experiment), predicts future nest material use in the absence of social information, and closely follows the methods of previous work [35,36,54,57,58].

If the male observer interacted with the string for a total duration of less than 30 seconds, the preference test was repeated daily until this minimum threshold was met. The majority of observer pairs (73/77) required only one initial colour preference test session to reach the 30-second threshold, with four pairs requiring two sessions. Initial colour preference tests were scored on the same day they were conducted. If the 30-second interaction threshold was reached (Mean = 3422 s, Range = 43–8683 s, SD = 2371 s), then the observer pair proceeded to Phase 2 of the experiment on the same day.

#### Phase 2: Social information phase.

**Fig 3:**
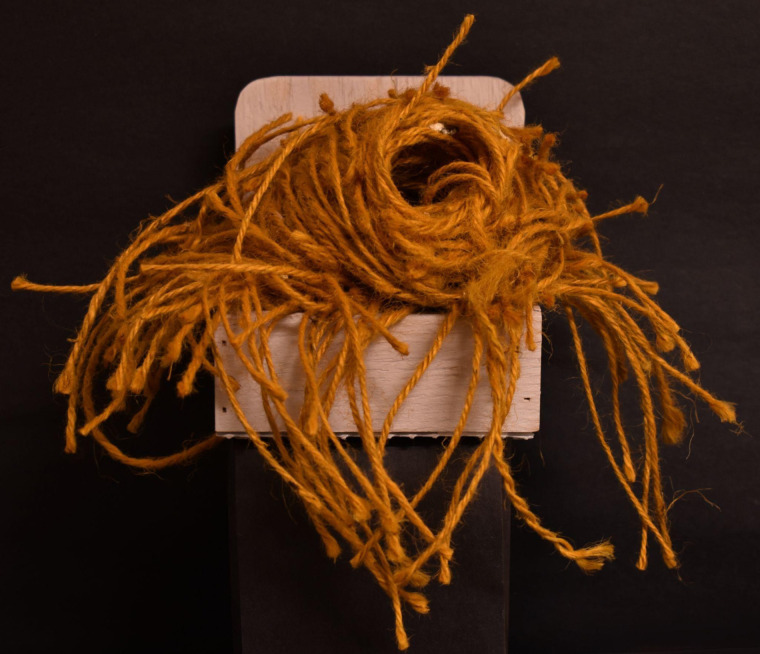
Front view of a yellow demonstrator nest. Each demonstrator nest was constructed in a white nest cup using 400 pieces of 15 cm string.

The social information phase began when the observer pair was moved from the holding room into the centre cage of the social information room (Fig 1) and ended after 35 daylight hours had elapsed (~2.5 days). Each population contained four demonstrator pairs, where each demonstrator pair was incubating eggs in a nest constructed from either yellow or blue string (Figs 1 and 3). During daylight hours of the observation phase, the observer pair had continuous visual access to each demonstrator pair and their nests.

#### Phase 3: Final colour preference test.

Immediately following the social information phase, observer pairs were returned to their holding room cage for their final colour preference test. Each pair of observers was given a white wooden nest cup, hung in the centre back of their holding-room cage, and 25 pieces of each colour of string (blue and yellow, 50 pieces total) with which to construct a nest. Each colour of string was placed on the same side of the cage as in the initial colour preference test (Fig 2). The final colour preference test ended once all 50 pieces of string had been deposited into the nest cup, or after a maximum of five days. Video of the final colour preference test was recorded using in-cage cameras, and behavioural scoring was completed using BORIS Version 8.24.1.

### Behavioural scoring

Behavioural scoring of the final colour preference test was blinded with respect to initial colour preference, preference strength, and population. The following behaviours during the final colour preference test were scored: the colour of material that the observer male first (1) touched, (2) picked up, and (3) deposited into the nest cup (up to the 25th deposit, see Table 2) [55]. Final colour preference tests were scored by two experimenters. To assess interrater reliability, 14 randomly selected final colour preference test videos (total hours = 95) were scored by both researchers who scored videos and Cohen’s kappa (κ) was calculated. κ is a chance-corrected measure of interrater reliability, with values interpreted as indicating none, slight, fair, moderate, substantial, or near-perfect agreement. In this study, all κ values were ≥ 0.75, indicating substantial interrater reliability.

**Table 2:** Definitions of Behaviours Scored During the Final Colour Preference Test.

Interaction Type	Definition
**Touch**	Contact of any body part with the material, excluding the tail.
**Pick-up**	Grasp and lift material from surface level to an elevated position using the beak.
**Deposit**	Material brought to and released into the nest cup.

### Data analysis

All data were analysed in R 4.4.1 [59]. All models were fit using the R package *stats* [59]. Model assumptions and fit were assessed using the R package *performance* [60]. Due to high collinearity between our ‘initial preference strength’ variable and interaction term (‘initial preference strength × ‘population’), we mean-centered the ‘initial preference strength’ variable in all four of our models.

#### Final sample size.

Two pairs were excluded from the analysis because they did not deposit any string into the nest cup within five days of starting the final colour preference test. The final sample size was n = 75 observer pairs, with n = 15 pairs in the 4P-0N population, n = 15 pairs in the 3P-1N population, n = 15 pairs in the 2P-2N population, n = 15 pairs in the 1P-3N population, and n = 15 pairs in the 0P-4N population.

#### First material interactions in the final colour preference test.

To determine the effect of the population on first material interactions during the final colour preference test, we specified three generalized linear models (GLMs), each with a binary dependent variable indicating whether the interaction was directed towards the observer male’s initially non-preferred colour (1 = yes, 0 = no). Separate models were constructed for the first material (1) touched, (2) picked up, and (3) deposited into the nest cup, following methods from [36]. Given the binary nature of the response variables, we used GLMs with a binomial distribution and a logit link function. Each model included three fixed effects: (1) population (n = 5), (2) initial preference strength (%), and (3) the interaction between population and initial preference strength. Initial preference strength was included as a predictor due to its role in influencing nest material use [54], and because both empirical studies and recent theoretical models suggest that individual preferences are important predictors of conformity [61].

#### Final colour preference test.

To determine the effect of the population on final colour preference, we specified a generalized linear model (GLM). The dependent variable for this model was the proportion of the non-preferred colour strings (out of the first 25) deposited during the final preference test. As in the three prior models, our fixed effects were: (1) population, (2) initial preference strength, and (3) the interaction between population and initial preference strength. As the response variable was proportional, we initially specified a binomial family for our model. Diagnostic checks indicated overdispersion, so we refit the model with a quasibinomial family. The quasibinomial family estimates a dispersion parameter and inflates standard errors to accommodate extra-binomial variation, yielding more conservative inference (reduced statistical power) than would be expected if the binomial variance assumption were met.

## Results

### Initial colour preference test

**Fig 4:**
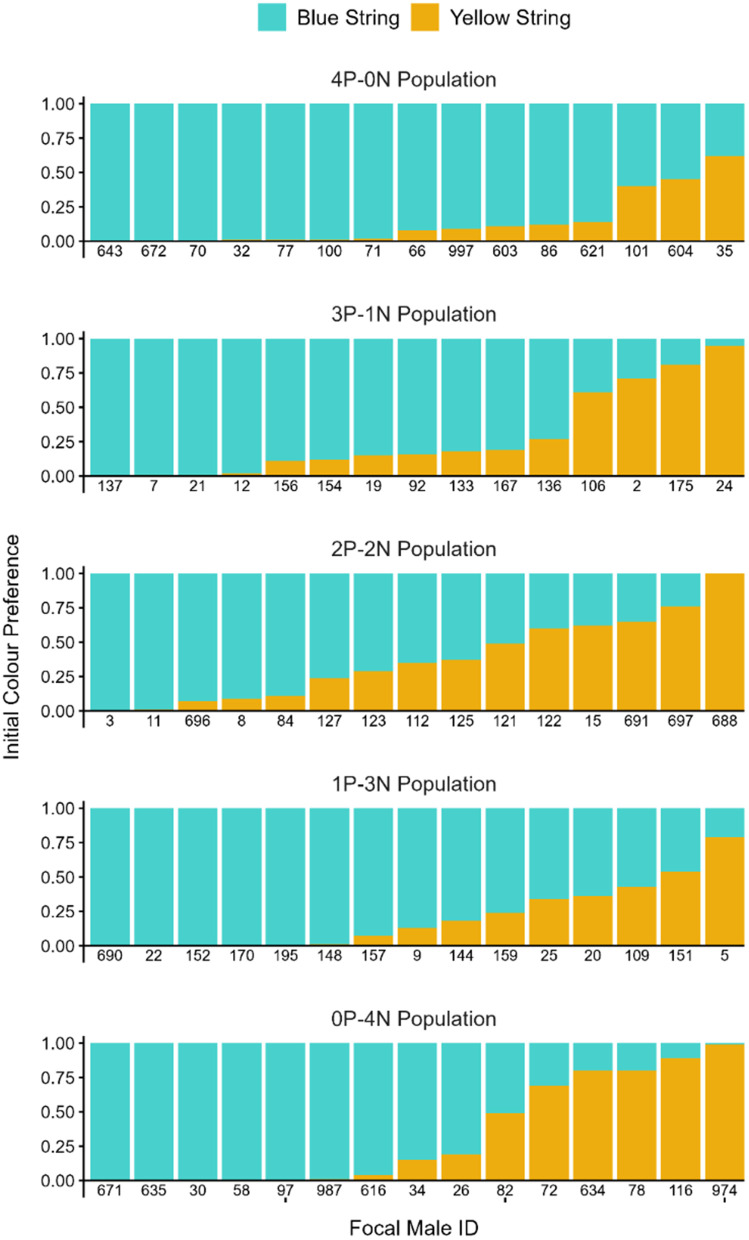
Stacked bar charts showing the initial colour preferences of each observer male, grouped and labelled by experimental population. Each bar represents one observer male, with the y-axis showing the proportion of total interaction time spent with each colour during the initial colour preference test. Blue bar segments show the proportion of time spent interacting with blue string, and yellow bar segments show the proportion of time spent interacting with yellow string.

Of the seventy-five observer pairs retained in the analysis, seventeen pairs preferred yellow, and fifty-eight preferred blue (see Table 3 for descriptive statistics; Fig 4).

**Table 3:** Descriptive Statistics for the Initial Colour Preference Test.

Population	Pairs Preferring Yellow	Pairs Preferring Blue	Mean Preference Strength	Standard Deviation	Median Preference Strength
4P-0N	1	14	88%	16%	92%
3P-1N	4	11	86%	12%	85%
2P-2N	5	10	77%	16%	76%
1P-3N	2	13	84%	12%	87%
0P-4N	5	10	89%	15%	96%

### First material interactions

#### Model 1: First touch.

There was a significant main effect of population composition on the first material touched during the final colour preference test (*β* = 0.512, *SE* = 0.257, *z* = 1.990, *p* = 0.047). As the number of nests built from the focal male’s initially non-preferred colour increased in the population, the probability that the male directed his first touch towards his initially non-preferred colour also increased. The main effect of initial preference strength was not statistically significant (*β* = 0.371, *SE* = 4.907, *z* = 0.075, *p* = 0.940), and the interaction between population composition and initial preference strength was also not statistically significant (*β* = −1.916, *SE* = 1.825, *z* = −1.050, *p* = 0.294).

#### Model 2: First pickup.

There was no statistically significant main effect of population composition (*β* = 0.384, *SE* = 0.225, *z* = 1.703, *p* = 0.089) or initial preference strength (*β* = −0.222, *SE* = 4.065, *z* = −0.055, *p* = 0.956) on the first material picked up during the final colour preference test. The interaction between population composition and initial preference strength was also not statistically significant (*β* = −1.666, *SE* = 1.575, *z* = −1.057, *p* = 0.290).

#### Model 3: First deposit.

There was no statistically significant main effect of population composition (*β* = 0.114, *SE* = 0.250, *z* = 0.454, *p* = 0.650) or initial preference strength (*β* = 1.832, *SE* = 4.357, *z* = 0.420, *p* = 0.674) on the first material deposited during the final colour preference test. The interaction between population composition and initial preference strength was also not statistically significant (*β* = −1.865, *SE* = 1.647, *z* = −1.132, *p* = 0.258).

### Nest building

#### Model 4: Final colour preference.

**Fig 5:**
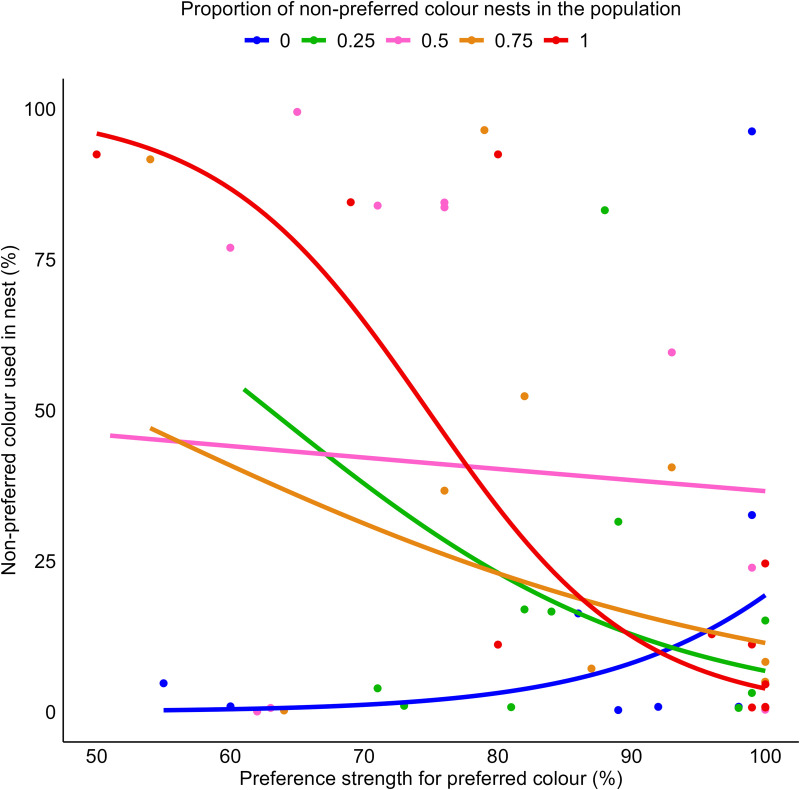
Relationship between initial preference strength and use of the initially non-preferred colour during the final colour preference test, plotted separately by population. The y-axis shows the percentage of the first 25 deposits made with the observer male’s initially non-preferred colour string. The x-axis shows the observer male’s initial preference strength. Each dot represents one observer male (colour-coded by the population he observed), and each line shows the fitted slope for that population. See S1 Fig for a version of this graph with 95% confidence intervals for each group.

There was no statistically significant main effect of either population composition (*β* = 0.154, *SE* = 0.172, *t* = 0.892, *p* = 0.375) or initial preference strength (*β* = 1.231, *SE* = 2.950, *t* = 0.417, *p* = 0.678) on the use of the non-preferred colour during the final colour preference test. The interaction, however, between population composition and initial preference strength was near-threshold significant (*β* = −2.410, *SE* = 1.214, *t* = −1.985, *p* = 0.051, Fig 5). As the number of nests built from the focal male’s non-preferred colour increased in the population, the probability that he used that colour in the final preference test also increased, but only when his initial preference was relatively weak. Males with stronger preferences were less likely to change their preference, regardless of which population they observed. This model was fit using a quasibinomial family to accommodate overdispersion (see ‘Data analysis’ section), which may reduce statistical power.

## Discussion

Our findings suggest that conformity in nest material use is moderated by the strength of individual preferences. Males with weaker preferences were more susceptible to social influence, conforming to the majority by constructing nests with their non-preferred material. Conversely, individuals with stronger preferences were less likely to use social information and instead selected materials that aligned with their own preferences. Majority behaviour of the population also influenced individuals to re-examine the socially demonstrated colour; birds were more likely to first interact with their non-preferred material when it matched the colour demonstrated by the majority. However, this effect diminished with the subsequent nest-building decisions, including the first material picked up and deposited into the nest. We did not find evidence of conformist transmission (see S1 Table). These results show that nesting decisions are shaped by an interaction between personal preference and the broader social environment, reflecting a balance between personal preferences and conformity to group norms.

Our study design allowed us to examine both conformity and conformist transmission while distinguishing between the *acquisition* and *use* of social information during an ecologically relevant behaviour. This distinction is crucial for interpreting instances of low or non-conformity: whether individuals failed to acquire social information or instead chose not to act on it. Our results indicate that individuals often attended to and acquired social information, as evidenced by the effect of population on the material birds touched during nest building. However, birds did not always incorporate this information into their nesting choices (i.e., the first material they touched or deposited into their nest). Instead, the likelihood of social information use was moderated by the strength of individual nest material preferences. Thus, we provide evidence for both the acquisition and selective use of social information, highlighting individual preference strength as a key factor influencing conformity to group norms.

These findings provide experimental support for field-based observations of social transmission of cultural behaviour in avian nesting decisions. In wild pied flycatchers (*Ficedula hypoleuca*), the incorporation of anthropogenic materials into nests varied markedly across study sites. This variation, however, was not explained by geographical features or proximity to human development, suggesting a role for social transmission in shaping nest construction decisions [62]. A similar pattern was observed in blue tits (*Cyanistes caeruleus*), where the amount of aromatic plant matter used in nests remained consistent within, but differed between, populations [39]. These population-level differences persisted across breeding seasons and were not fully accounted for by differences in individual material preferences or availability of local vegetation, again suggesting a potential role for social transmission and local traditions in nest material use.

Evidence of social influence in nesting decisions has similarly been reported in species that do not construct their own nests. For example, female brood-parasitic brown-headed cowbirds (*Molothrus ater*) who lacked personal information about the quality of host nests were more likely to copy the choices of knowledgeable conspecifics, spending more time prospecting nests that were previously visited by others. In contrast, females with access to up-to-date personal information relied on their own assessments when choosing egg-laying sites [32]. This conditional use of social versus personal information is echoed in house-hunting ants (*Myrmecina nipponica*), where nest substrate selection depended both on the strength of individual preference and social conformity. When ants held weak preferences for nest substrates, they conformed to the nest substrate choices of their nest-mates; however, when their preferences were strong, ants relied on their own assessments, even when these conflicted with available social information [63].

The relationship between preference strength and conformity is well established in the human psychological literature: individuals with strong pre-existing preferences, attitudes, or beliefs, are generally less susceptible to social influence [64–67]. This resistance to social influence is partly explained by *motivated cognition* (also called positive confirmation bias), a cognitive bias in which people selectively interpret information in ways that support their preferred views [68–70]. For example, in a U.S. study, participants identifying as either Republican or Democrat were presented with identical, objective information about the anticipated impacts of climate change on public health. Despite being exposed to the same information, each group interpreted this information through the lens of their political affiliation, resulting in increased polarization: Democrats became more supportive of climate change mitigation efforts, whereas Republicans became *less* supportive of these same policies [71]. These findings, together with those of the present study, suggest that strong individual preferences can act as a filter through which social information is interpreted and serve as a buffer against social influence, reducing conformity to majority behaviours in multiple species, from ants, to birds and humans.

To date, social influence, particularly in the form of conformity and conformist transmission, has been examined across a wide range of species and behavioural contexts, from foraging in fish [26,72], birds [16,73], primates [24,25,74], and rats [75], to spatial navigation in dogs [76], and mate choice in fruit flies [11]. These studies have identified numerous factors that influence conformity, such as the size of the population majority and the relative benefits of majority behaviour. Additionally, several previous studies suggest a role of individual preference in shaping susceptibility to social influence [32,54,63]. Here, we contribute to this body of research by examining conformity in a novel and ecologically important context: avian nest building. By accounting for the strength of individual preferences in our models, we demonstrate that conformity to group norms is moderated by the strength of individual preferences.

Given that conformity plays a key role in the emergence and stability of culture, individual variability in preference strength may be critical for predicting where cultural traditions are likely to form and persist. Future research examining social learning and cultural dynamics should therefore consider the role of, and account for, individual preferences. Given the results from this study and others suggesting a role for socially influenced nest material use [35,39,41,54,62], broader experimental investigations examining other nest-building decisions (e.g., amount of material used, size/shape of the nest, location of the nest, nest-building initiation) would be useful for determining which nest-building decisions and outcomes are shaped by the social environment. For instance, results from recent fieldwork suggest a role for social influence in shaping nest morphology, so this is a promising area for future experimental work [38].

## Supporting information

S1 Table Expected versus Actual Results for Conformity and Conformist Transmission. (DOCX)

S1 Fig Relationship between initial preference strength and use of the initially non-preferred colour during the final colour preference test, with 95% confidence intervals and plotted separately by population. The y-axis shows the percentage of the first 25 deposits made with the observer male’s initially non-preferred colour string. The x-axis shows the observer male’s initial preference strength. Each dot represents one observer male (colour-coded by the population he observed), and each line shows the fitted slope for that population.
(PNG)

S1 Video Top-down view of an Initial Colour Preference Test. (MP4)

S2 Video Front-on view of an observer male depositing material into a nest cup during a Final Colour Preference Test. (MP4)

S1 Data Excel file of the data presented in this paper. (XLSX)

S1 R Code R code of the statistical analyses presented in this paper. (PDF)
