# Supplementary material for: Conformity and individual preference shape nest material use in zebra finches (Taeniopygia guttata)
Source: PLoS One. 2026 Feb 11;21(2):e0342277. doi: 10.1371/journal.pone.0342277 (PMC12893555; doi:10.1371/journal.pone.0342277)
Supplement: S1 Table — (DOCX) [file pone.0342277.s001.docx]

|  | **Expected Results if Proportional Copying** | | **Expected Results if Conformist Transmission** | | **Actual Data** | |
| --- | --- | --- | --- | --- | --- | --- |
| **Population** | **Nonpreferred Colour Deposits (%)** | **Observers Who Built Primarily with the Nonpreferred Colour (%)** | **Nonpreferred Colour Deposits (%)** | **Observers Who Built Primarily with the Nonpreferred Colour (%)** | **Nonpreferred Colour Deposits (%)** | **Observers Who Built Primarily with the Nonpreferred Colour (%)** |
| **4P-0N**  *100% preferred colour* | 0% | 0% | 0% | 0% | 10.5% | 7% |
| **3P-1N**  *75% preferred colour* | 25% | 25% | <25% | <25% | 19.4% | 13% |
| **2P-2N**  *50% preferred colour / 50% nonpreferred colour* | 50% | 50% | ~50% | ~50% | 40.8% | 47% |
| **1P-3N**  *75% nonpreferred colour* | 75% | 75% | >75% | >75% | 22.4% | 20% |
| **0P-4N**  *100% nonpreferred colour* | 100% | 100% | 100% | 100% | 22.1% | 20% |
| *Note:* To show conformist transmission, the proportion of observers matching the majority preference must be *significantly greater* than the proportion of group members demonstrating that preference (e.g. If 75% of demonstrators in the population built blue nests, significantly greater than 75% of observers would need to build blue nests). | | | | | | |
