# Supplementary figures and images for: Conformity and individual preference shape nest material use in zebra finches (Taeniopygia guttata)
Source: PLoS One. 2026 Feb 11;21(2):e0342277. doi: 10.1371/journal.pone.0342277 (PMC12893555; doi:10.1371/journal.pone.0342277)

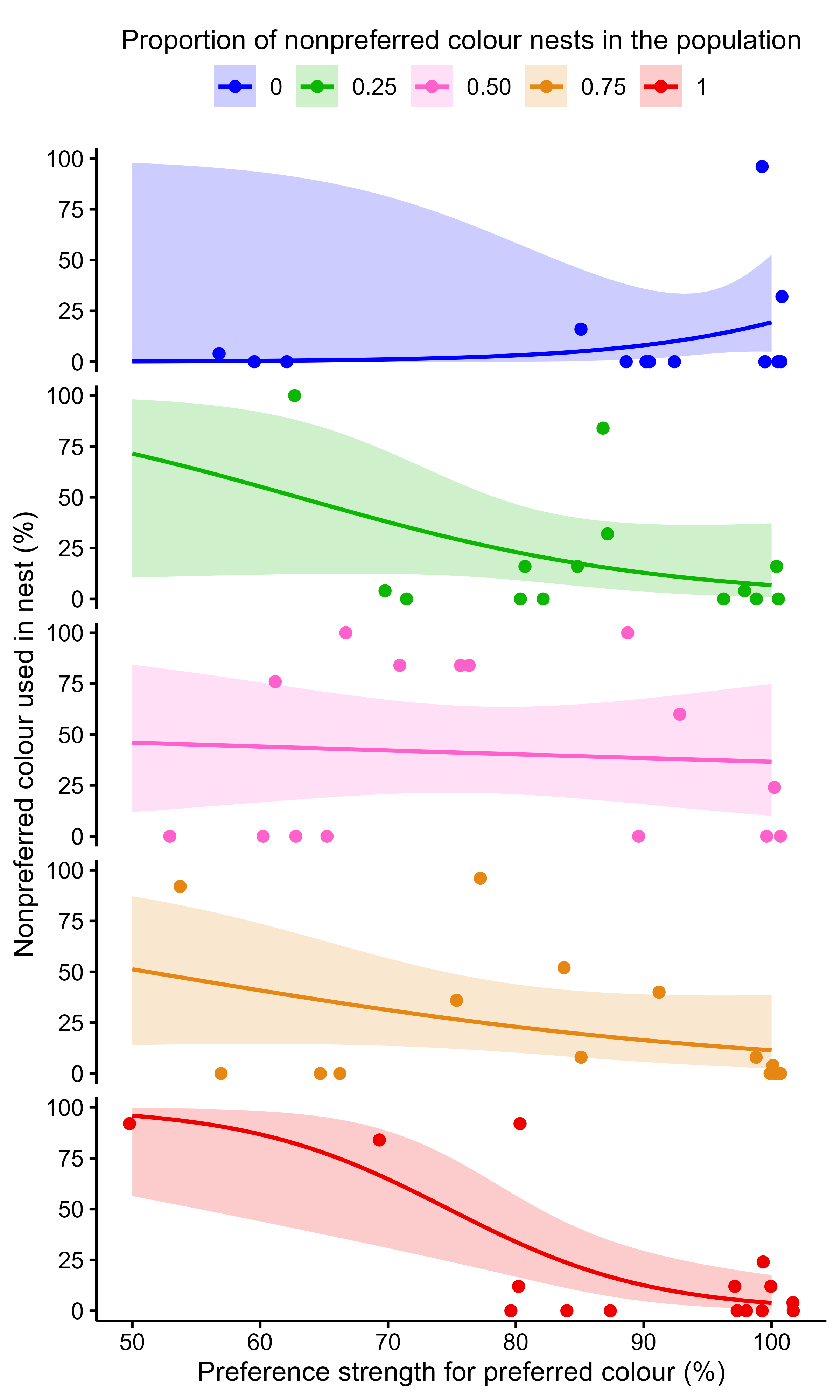

Supplement: S1 Fig — The y-axis shows the percentage of the first 25 deposits made with the observer male’s initially non-preferred colour string. The x-axis shows the observer male’s initial preference strength. Each dot represents one observer male (colour-coded by the population he observed), and each line shows the fitted slope for that population. (PNG) [file pone.0342277.s002.png]
