# Supplementary material for: Conformity and individual preference shape nest material use in zebra finches (Taeniopygia guttata)
Source: PLoS One. 2026 Feb 11;21(2):e0342277. doi: 10.1371/journal.pone.0342277 (PMC12893555; doi:10.1371/journal.pone.0342277)
Supplement: S1 R Code — (PDF) [file pone.0342277.s006.pdf]

```r
#### Conformity and individual preferences shape nest material use in zebra finches  ####
#### Load packages ####
library(performance)
library(ggplot2)
library(dplyr)
library(stats)

#### Load model data ####
data <- read.csv("Data.csv")

### Model 1: First touch model ###
First_Touch_Model <- glm(
  First_Touch ~ Number_Nonpreferred_Nests * IPT_Strength_centered,
  family = binomial(link = "logit"),
  data = subset(data, !is.na(First_Touch))
)
# Check model fit
summary(First_Touch_Model)
performance::check_model(First_Touch_Model)

### Model 2: First pick-up model ###
First_Pickup_Model <- glm(
  First_Pickup ~ Number_Nonpreferred_Nests * IPT_Strength_centered,
  family = binomial(link = "logit"),
  data = subset(data, !is.na(First_Pickup))
)
# Check model fit
summary(First_Pickup_Model)
performance::check_model(First_Pickup_Model)

### Model 3: First deposit model ###
First_Deposit_Model <- glm(
  First_Deposit ~ Number_Nonpreferred_Nests * IPT_Strength_centered,
  family = binomial(link = "logit"),
  data = subset(data, !is.na(First_Deposit))
)
# Check model fit
summary(First_Deposit_Model)
performance::check_model(First_Deposit_Model)

### Model 4: Material use model ###
Material_Use_Model <- glm(
  cbind(Count_Nonpreferred, Count_Preferred) ~ IPT_Strength_centered *
Number_Nonpreferred_Nests,
  family = quasibinomial,
  data = subset(data, !is.na(Count_Nonpreferred) & !is.na(Count_Preferred))
)
# Check model fit
summary(Material_Use_Model)
performance::check_model(Material_Use_Model)
```
